# Hourglass Sign in Liver Hydatid Disease: Significance and Therapeutic Implications

**DOI:** 10.4269/ajtmh.23-0722

**Published:** 2024-04-16

**Authors:** Anis Hasnaoui, Racem Trigui, Firas Attig

**Affiliations:** ^1^ Department of General Surgery, Menzel Bourguiba Hospital, Bizerta, Tunisia;; ^2^ Faculty of Medicine of Tunis, Tunis El Manar University, Tunis, Tunisia

**Figure 1.**
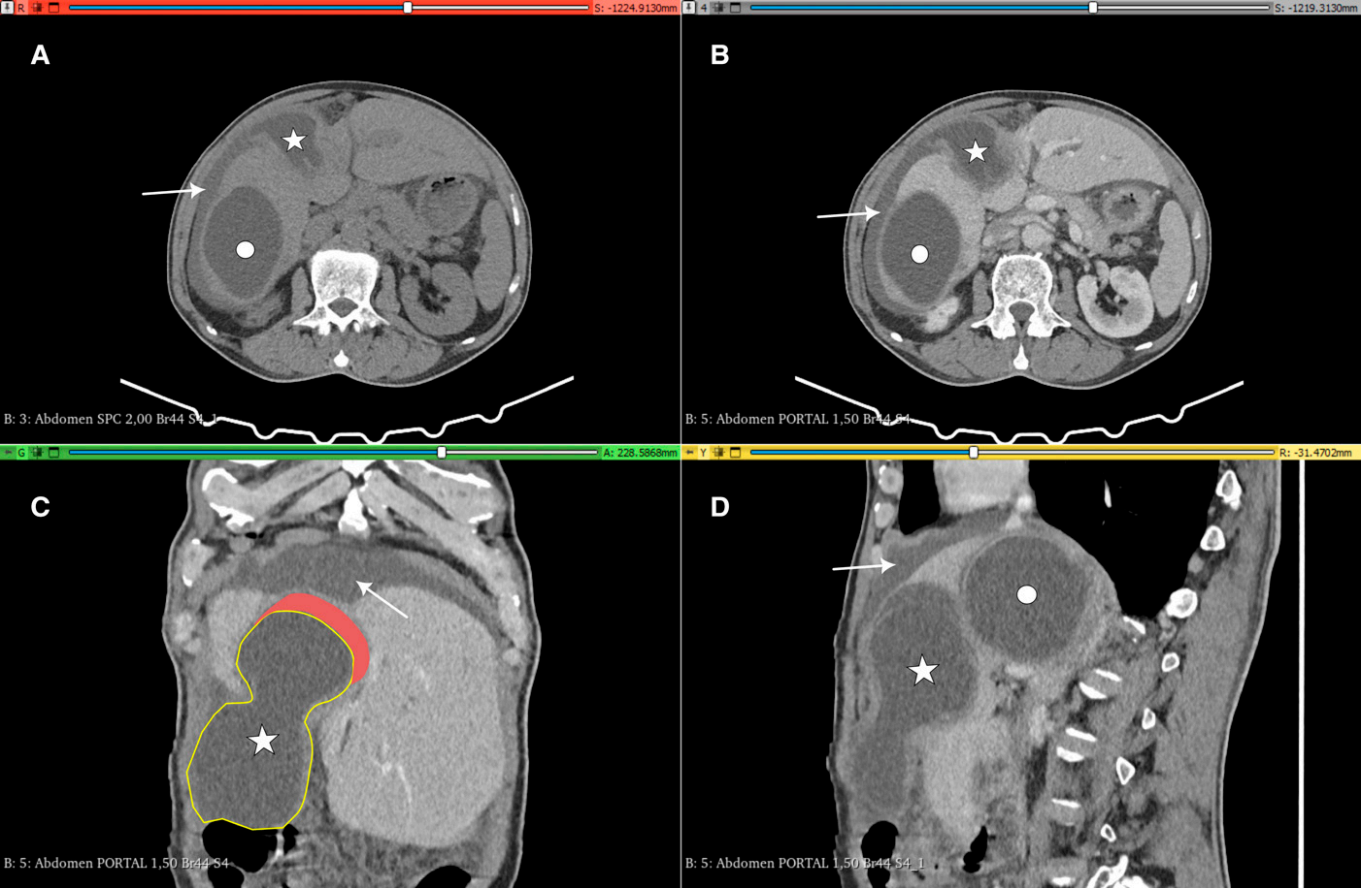
Preoperative computed tomography scan. (**A**) Nonenhanced axial view, (**B**) portal phase axial view, (**C**) portal phase coronal view, and (**D**) portal phase sagittal view, showing two hydatid cysts in the liver (white circle and white star). Perihepatic effusion is indicated by white arrows. The hourglass shape is depicted by a yellow contour in panel C. The cleavage plane between the laminated membrane and the pericyst is highlighted in red in Panel C.

**Figure 2.**
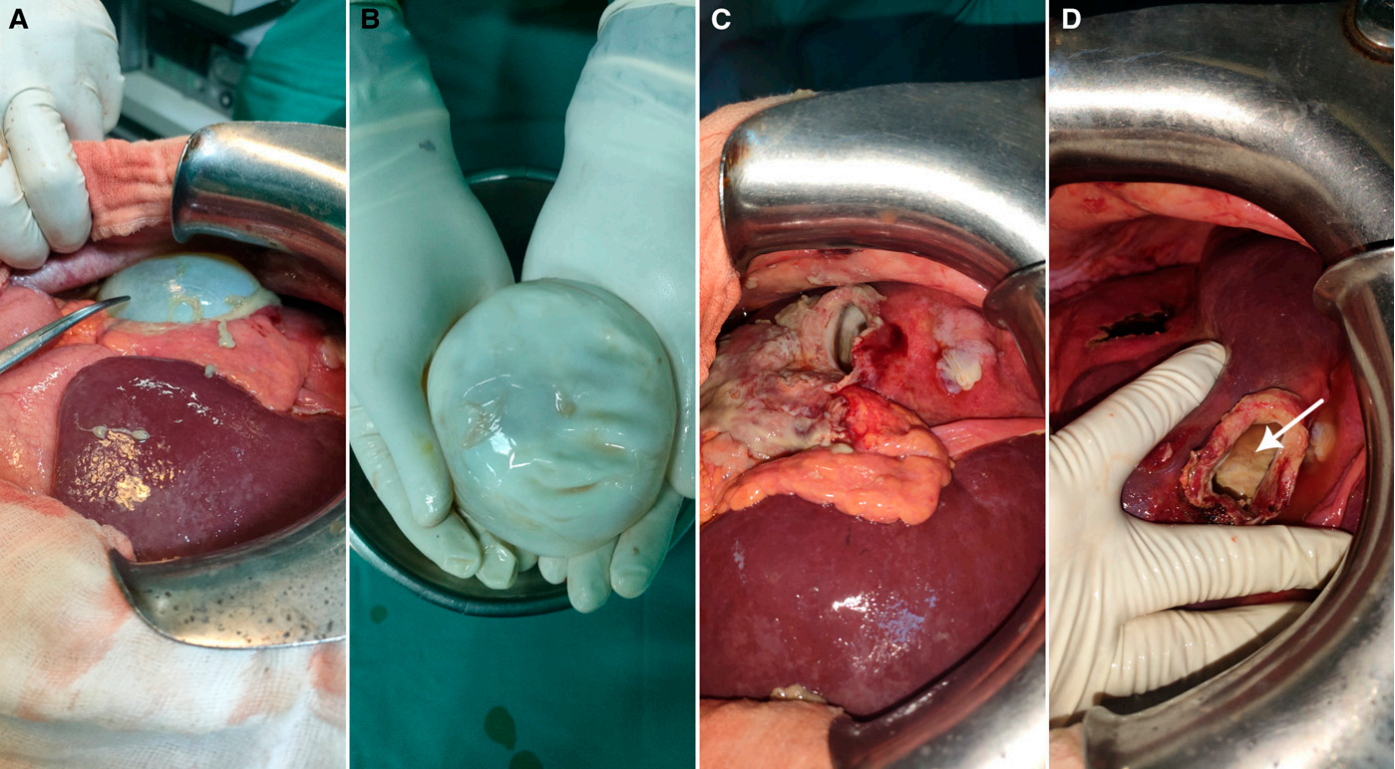
Intraoperative findings. (**A**) A laminated membrane of a hydatid cyst protruding beyond the liver’s surface. (**B**) The delivered hydatid cyst with an intact laminated membrane. (**C**) Burst pericyst. (**D**) A discernible whitish coating in the residual cavity, denoting sclerotic alterations (white arrow).

A 57-year-old sheep breeder was admitted to our surgery department for pain in the right hypochondrium. Physical examination and laboratory findings were normal. Notably, there were no indications of fever, jaundice, or signs of an allergic reaction. Computed tomography imaging showed two subcapsular hydatid cysts in the hepatic dome (Figure 1). The first was a noncomplicated type 1 cyst according to the classification of the WHO. The second cyst exhibited a distinctive hourglass shape, as depicted in Figure 1C, with a portion of it protruding beyond the liver’s surface (Figure 1C and D). There was also a cleavage plane between the laminated membrane and the pericyst (Figure 1C). Intraoperative findings confirmed the partial migration of an intact hydatid cyst (Figure 2A). After the delivery of the cyst with an intact laminated membrane (Figure 2B), the pericyst was inspected (Figure 2C and D). It displayed a whitish coating, indicating sclerotic alterations (Figure 2D). No sign of biliary fistula was found.

Ultimately, conservative surgical management was performed for the remaining cyst. The pathology report confirmed the hydatid nature of the cysts. The postoperative course was uneventful, and the patient was initiated on 400 mg of albendazole twice a day for 3 months. The hourglass sign is an imaging sign referring to a waist-like constriction of a herniated structure through a defect.^1^ In liver hydatid disease, trans-diaphragmatic migration of hydatid cysts located in the hepatic dome could display this sign.^2^ This migration is facilitated by the pressure difference between the thorax and the abdomen. In our case, this sign referred to a pericyst rupture and partial expulsion of a hydatid cyst in the peritoneal cavity with intact laminated membrane. Unlike trans-diaphragmatic migration, the expulsion of an intact laminated membrane in the peritoneal cavity is an exceptionally rare occurrence. The pericyst is a fibro-conjunctive shell resulting from a sclerotic response generated by the host. As the cyst expands in size, the resulting compression on the liver parenchyma gradually weakens the pericyst due to fibrosis and ischemic processes, leading to degeneration and eventually a spontaneous expulsion of the cyst.^3^ Management of this condition is exclusively surgical. It is an emergency due to the risk of secondary membrane rupture, potentially causing anaphylactic shock. Puncture, aspiration, injection, reaspiration (known as PAIR)^4^ is not feasible because it could result in total migration of the hydatid cyst into the peritoneal cavity after the aspiration of its contents.
